# Secondary Lymphoid Organs in Mesenchymal Stromal Cell Therapy: More Than Just a Filter

**DOI:** 10.3389/fimmu.2022.892443

**Published:** 2022-06-16

**Authors:** Di Zheng, Tejasvini Bhuvan, Natalie L. Payne, Tracy S. P. Heng

**Affiliations:** ^1^ Department of Anatomy and Developmental Biology, Biomedicine Discovery Institute, Monash University, Clayton, VIC, Australia; ^2^ Australian Regenerative Medicine Institute, Monash University, Clayton, VIC, Australia; ^3^ ARC Training Centre for Cell and Tissue Engineering Technologies, Monash University, Clayton, VIC, Australia

**Keywords:** mesenchymal stromal cells, cell therapy, immune responses, apoptosis, secondary lymphoid organs, spleen, lymph nodes, efferocytosis

## Abstract

Mesenchymal stromal cells (MSCs) have demonstrated therapeutic potential in inflammatory models of human disease. However, clinical translation has fallen short of expectations, with many trials failing to meet primary endpoints. Failure to fully understand their mechanisms of action is a key factor contributing to the lack of successful commercialisation. Indeed, it remains unclear how the long-ranging immunomodulatory effects of MSCs can be attributed to their secretome, when MSCs undergo apoptosis in the lung shortly after intravenous infusion. Their apoptotic fate suggests that efficacy is not based solely on their viable properties, but also on the immune response to dying MSCs. The secondary lymphoid organs (SLOs) orchestrate immune responses and play a key role in immune regulation. In this review, we will discuss how apoptotic cells can modify immune responses and highlight the importance of MSC-immune cell interactions in SLOs for therapeutic outcomes.

## Introduction

Mesenchymal stem cells, more accurately known as mesenchymal stromal cells (MSCs), are one of the most widely investigated therapeutic cell types, owing to their ease of accessibility and expansion from tissues free of ethical concerns, as well as their immunomodulatory capacity in various preclinical disease models (1–3) . MSCs can be sourced from the stroma of almost all tissues, but most commonly from bone marrow (BM), adipose tissue (AD) and umbilical cord (UC) (4, 5). MSCs are contained within a heterogeneous population expressing CD105, CD73 and CD90 while lacking CD45, CD11b, CD19 and HLA-DR. They are characterized by their adherence to plastic and ability to differentiate into osteoblasts, adipocytes and chondrocytes *in vitro* (6).

With increasing clinical translation, however, this minimal set of criteria is now recognized as inadequate for defining MSCs. It does not reflect MSC potency, which is largely based on broad immunomodulatory properties (7), rather than their self-renewal or multipotential capacity (8, 9). It does not address changes in marker expression levels or biological properties due to culture expansion and cell manufacturing processes (10), including cryopreservation and freeze-thawing (11). Additionally, it does not identify the risk profile of the MSC product, particularly with regard to hemocompatibility of intravenously (IV) delivered cells and their potential to trigger adverse thromboembolic events (12). Thus, proposed amendments to the criteria have included functional potency assays based on the immunomodulatory activity of MSCs (13), and profiling of the hemocompatibility of the diverse array of MSC products now in clinical use (14).

**Figure 1:**
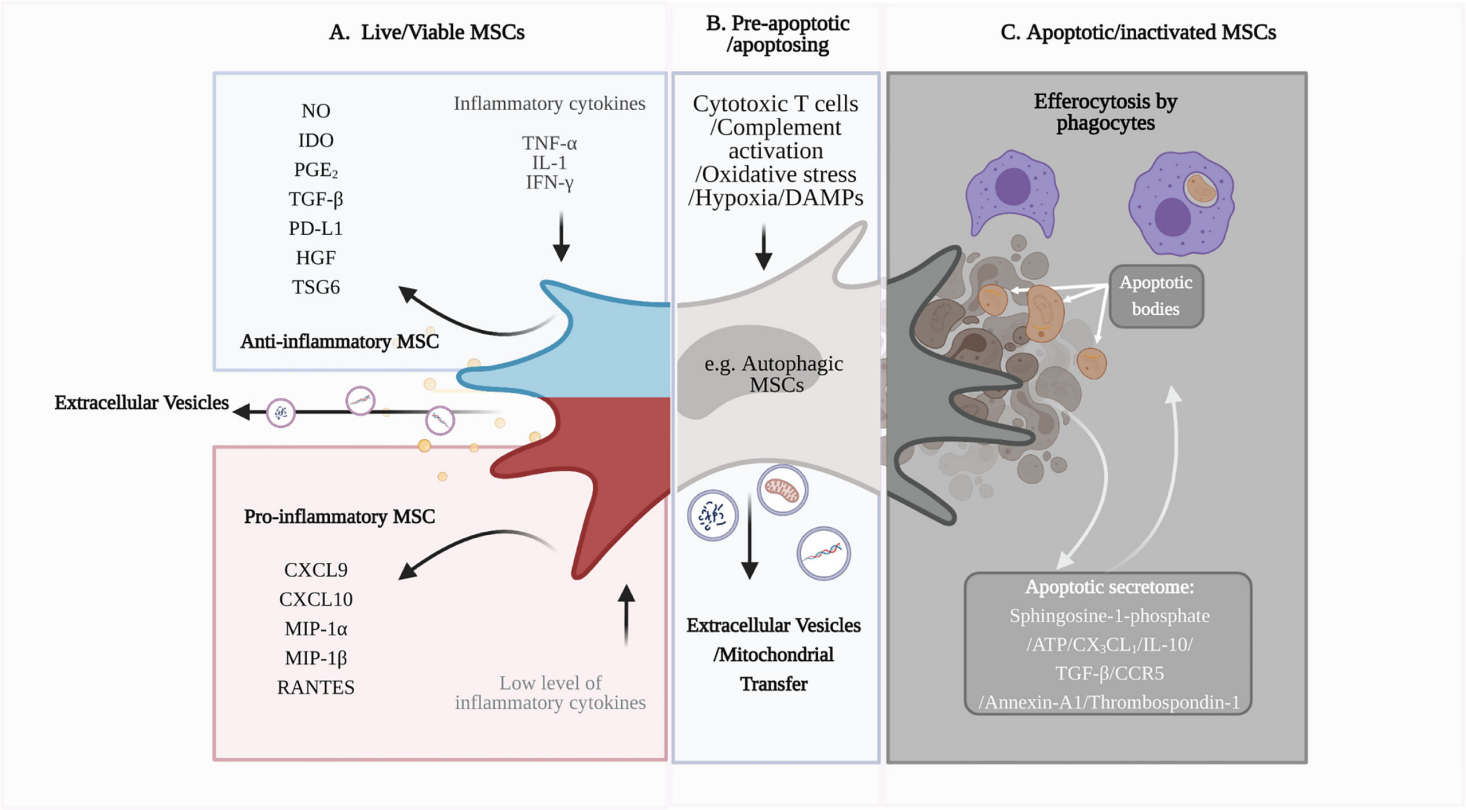
Immunomodulatory capacity of viable and apoptotic MSCs. **(A)** Live or viable MSCs can sense the microenvironment and respond to cytokine signals by polarizing into ‘pro-inflammatory’ or ‘anti-inflammatory’ phenotypes (24). ‘Anti-inflammatory’ MSCs produce anti-inflammatory soluble factors, including IDO and TGF-β, to modulate immune cell function and dampen inflammation. MSCs also produce extracellular vesicles (EV), such as exosomes, that can be immunomodulatory, depending on their cargo (19, 25). **(B)** Excessive inflammatory stress or the presence of cytotoxic immune cells will induce apoptosis of MSCs (26). MSCs can undergo a pre-apoptotic stage, known as autophagy. Autophagic MSCs, as well as the EVs produced during this stage, may have roles in immunosuppression (27). **(C)** As MSCs undergo apoptosis, their apoptotic secretome can promote an anti-inflammatory microenvironment and attract phagocytes for efferocytosis (28). Phagocytes that have engulfed the apoptotic MSCs become immunomodulatory and have downstream regulatory effects on adaptive immune cells, such as T cells (29). Figure was created with BioRender.com.

The preclinical efficacy of MSCs in various unrelated conditions such as graft-versus-host disease (GvHD), Crohn’s disease, kidney transplantation, myocardial infarction, stroke, diabetes, acute respiratory distress syndrome (ARDS), multiple sclerosis, and brain and spinal cord injury, mainly relates to immune regulation (15–20). *In vitro* studies have shown that MSCs can modulate adaptive and innate immune responses. MSCs suppress T cell proliferation, cytokine response (e.g. IFN-γ production) and cytotoxic activity in response to antigen-specific stimuli (21–23) whist promoting regulatory T cells (Tregs), *via* their production of soluble factors (**Figure 1**) such as nitric oxide (NO), indolamine 2,3 dioxygenase (IDO) and transforming growth factor-beta (TGF-β) (24, 30–32). MSCs can also downregulate the cytokine responses of innate immune cells, including dendritic cells (DCs) and monocytes, *via* the expression of prostaglandin E2 (PGE_2_) (33). How these *in vitro* findings relate to their mode of action remains to be clarified, given the complexity of immune responses *in vivo* and the short persistence of MSCs following infusion.

## MSC Survival and Biodistribution

To date, studies have investigated different routes of MSC administration in order to maximise therapeutic efficacy. The different administration routes result in variations in MSC survival. IV infusion has been the most commonly used and studied method for MSC delivery because it is convenient, minimally invasive and reproducible (4). However, MSCs administered *via* the IV route are entrapped in the lung and rapidly cleared, with few traces detected in other tissues (34, 35). Studies using various MSC detection techniques, including MSCs constitutively expressing fluorescent proteins or luciferase, or labelled with fluorescent dyes or radioactive tracers, showed that viable MSCs are detected in the lung within 24 hours of IV administration but not at 72 hours (35–37). The decrease in detectable viable cells is directly proportional to the increase in dead cells in the lungs, indicating that IV-infused MSCs undergo cell death (37). Activation of caspase 3, a hallmark of apoptosis, in MSCs further indicates that MSCs undergo programmed cell death following their entrapment in the lung (38, 39).

The survival of IV-delivered MSCs can also be compromised if cells trigger the instant blood-mediated inflammatory reaction (IBMIR) due to incompatibility with blood (40). Expression of secreted and cell surface immunogenic factors (e.g. tissue factor (TF)/CD142) vary across MSC tissue sources and cell manufacturing conditions, including freeze-thawing and culture passaging (12, 14). These factors can activate the innate immune system and trigger the coagulation and complement cascades, which limit MSC engraftment and efficacy, but also increase the potential for adverse thromboembolic events (41).

Other injection routes, including intraperitoneal (IP), subcutaneous (SC), and intramuscular (IM), which bypass the lung, have also been examined. Prolonged detection of MSCs has been observed following IP and SC injection (42, 43). Following IM injection, a dwell time of up to 5-month was observed (44). Other studies have administered MSCs directly into the diseased tissue, for example, intratracheal administration in models of lung inflammation or intrathecal administration in models of spinal cord injury or neuroinflammatory disease (45–48). In these studies, MSCs are directly exposed to an inflammatory environment, which can influence MSCs survival and therapeutic efficacy (26, 49). Pre-conditioning of MSCs *via* hypoxia or serum deprivation for instance, can promote cell survival when subsequently exposed to an ischemic environment (50–52). Although the quantity, duration and the type of stress insult matter, overall, excessive stress will likely predispose MSCs to cell death (26).

In some disease settings, dead or dying MSCs and their associated ‘by-products’ can contribute to therapeutic efficacy (38, 39, 53–55) raising additional questions about their mode of action. How are MSCs killed in different microenvironments? How does the dying process contribute to therapeutic efficacy in different disease settings? Importantly, how do IV administered MSCs dying in the lung exert anti-inflammatory effects in distal organs in disease settings that seemingly do not involve the lung?

## MSC Apoptosis and Their Immunosuppressive ‘By-Products’

The molecular pathway that induces the death of MSCs *in vivo* is unclear and complicated by pre-existing disease, inflammatory cell infiltrate and the presence of different pathogens (26). The stress signals from the inflammatory microenvironment can trigger the apoptosis of MSCs. In a mouse model of GvHD, the presence of elevated numbers of cytotoxic CD8^+^ T cells and CD56^+^ natural killer (NK) cells in the lung caused MSC apoptosis (38). Settings that do not involve cytotoxic cell infiltrate likely involve other mechanisms. We recently showed that disabling the intrinsic (mitochondrial) pathway of apoptosis in MSCs prevented caspase 3 activation in the lung shortly after IV injection, indicating that MSCs were predominantly killed *via* the intrinsic pathway in non-GvHD settings (39). Activation of coagulation and complement by infused MSCs has also been shown to damage and reduce viability of MSCs (40, 56). Stronger activation of these proteolytic cascades is demonstrated by freeze-thawed (without culture recovery) and high-passage MSCs compared with fresh culture-derived, minimally expanded cells. This is due to variations in expression of immunogenic triggers, which may subsequently impact their *in vivo* therapeutic function (11).

In general, apoptosis is an ordered event that creates a transient immunosuppressive microenvironment *via* the release of anti-inflammatory mediators, including IL-10, TGF-β, CCR5, annexin-A1 and thrombospondin-1 (**Figure 1**) (28). Besides these secreted factors, cells can undergo a pre-apoptotic stage known as autophagy when they sense danger or stress signals from the microenvironment (57). By culturing MSCs in a stressed environment, autophagic MSCs can be pre-engineered to secrete immunomodulatory factors, such as TGF-β, to regulate T cell proliferation (58). Interestingly, MSCs have been shown to communicate with damaged cells *via* bidirectional transfer of mitochondria to increase mitochondrial biogenesis and rescue the cellular function of damaged cells (27). In preclinical models of myocardial infarction and respiratory disorders, mitochondrial transfer has been shown to contribute to the therapeutic effects of MSCs (**Figure 1**) (27, 59–61). MSCs can transfer mitochondria to macrophages *via* tunnelling nanotubules, cell fusion or extracellular vesicles (EV), which influence the macrophage function to modulate inflammation (59, 62). Whilst mitochondrial transfer has yet to be demonstrated in apoptotic MSCs, apoptotic bodies, which are a distinct type of extracellular vesicles formed when apoptotic cells disassemble into fragments (63), may also contribute to therapeutic efficacy upon engulfment by macrophages (64). Thus, MSCs are susceptible to cell death post-administration, which contributes to the anti-inflammatory effects of MSC therapy (10, 39).

## Efferocytosis of MSCs

Rapid clearance of apoptotic debris by phagocytes is essential in maintaining body homeostasis. Known as efferocytosis, this physiological process ‘silently’ removes apoptotic cells, inducing peripheral tolerance and avoiding inflammation. Phagocytic cells, including macrophages and monocytes, play a critical role in MSC therapy, as demonstrated in disease models where depletion of macrophages with clodronate liposomes renders MSC therapy ineffective (33, 36, 38). Phagocytes that have engulfed apoptotic cells, including MSCs, display a regulatory phenotype characterized by upregulation of PD-L1, IDO, COX2 and CD206, increased production of IL-6, IL-10, TGF-β and PGE_2_, and decreased production of pro-inflammatory cytokines such as TNF-α and IL-12 (29, 37, 65). Phagocytes that have engulfed apoptotic MSCs can suppress T cell proliferation, downregulate CD4^+^ T cell activation and promote Foxp3^+^ Treg generation (29, 37). The inhibition of COX2 abrogated the immunosuppressive capacity of efferocytic monocytes that had engulfed apoptotic MSCs, highlighting the importance of the PGE_2_/COX2 axis in this immunosuppressive function (29).

Since MSC apoptosis and the subsequent response of immune cells to this process contributes to their immunomodulatory effects, an outstanding question is whether viable MSCs are still required for MSC therapy, or can pre-inactivated MSCs be used as an alternative?

## Apoptotic or Dead MSCs as a Therapeutic Cell Option

Several studies have investigated the efficacy of *in vitro* induced apoptotic, dead or inactivated MSCs. Treatment outcomes can be influenced by the type and duration of stimulation used to inactivate the cells, and also the disease setting (26). Apoptotic MSCs (39, 53, 54), but not dead MSCs (36, 66) could inhibit lung inflammation. In certain disease settings, such as LPS-induced sepsis, inactivated or dead MSCs could replicate the immunomodulatory effects, as pre-inactivation enhanced the phagocytosis of MSCs (55, 67). In other settings, non-viable MSCs were ineffective or did not fully replicate the effects of viable MSCs (33, 42, 67). For instance, in GvHD, apoptotic MSCs had to be administered at a much higher dose than viable MSCs to achieve comparable therapeutic benefits (38). It is plausible that inflammatory diseases driven by innate immune or phagocytic cells are more likely to benefit from treatment with inactivated MSCs, while suppression of T cell responses require MSCs with an active secretome (67). Importantly, the type and stage of cell death at the time of MSC administration are key considerations, as apoptosis, but not necrosis or lytic cell death, induces anti-inflammatory responses (68).

Although MSC apoptosis and the subsequent host phagocytic response contribute to immunomodulation, there remains much to learn about the full mechanisms of MSC therapy. As MSC apoptosis and efferocytosis occurs in the lung post-infusion, how does this dampen inflammation in other organs or tissues? *In vitro* studies have shown that MSCs can regulate immune cell function *via* their paracrine activity, but such molecules must have a relatively long half-life and broad biodistribution for this to be a plausible mechanism *in vivo*. Immune responses are initiated and maintained in the SLOs, which play a key role in immune regulation during health and disease. Thus, our knowledge of how MSC therapy modulates immune responses would be improved by considering the known function of SLOs, their role in clearance of apoptotic cells and gaining a better understanding of the effects of MSCs in these organs.

## Organization and Function of Secondary Lymphoid Organs

Primary lymphoid organs, also known as central lymphoid organs, are the sites for the development and maturation of leukocytes. Primary lymphoid organs include the bone marrow and thymus (69–71). Lymphocytes (a class of leukocytes, e.g. T and B cells) generated in primary lymphoid organs then seed the SLOs where they initiate adaptive immune responses. SLOs include lymph nodes (LNs), spleen, tonsils, adenoids, Peyer’s patches, and mucosal-associated lymphoid tissues (MALT) (71). The localisation of lymphocytes in SLOs maximizes their interaction with foreign antigens that drain to the SLOs *via* blood or lymphatics. Antigen presenting cells, such as DCs, can also transport antigens to SLOs and activate lymphocytes there. Once activated, these lymphocytes undergo expansion and differentiate into effector or memory cells to provide antigen-specific responses. This review will focus on the spleen and LNs as the major SLOs.

### Structure and Function of the Spleen

The spleen is a network of branching arterial vessels that functions to filter blood, allowing for the capture of blood-borne pathogens and antigens, and is a key organ for iron metabolism and erythrocyte homeostasis (72, 73). Although early research suggested that excision of the spleen does not largely impact the human immune system, its functional significance has been demonstrated through various studies over the years. While the human and mouse spleen is similar in terms of gross structure and immune cell function, some key differences are known to exist and have been reported elsewhere (73–75). This section will focus on the mouse spleen.

**Figure 2:**
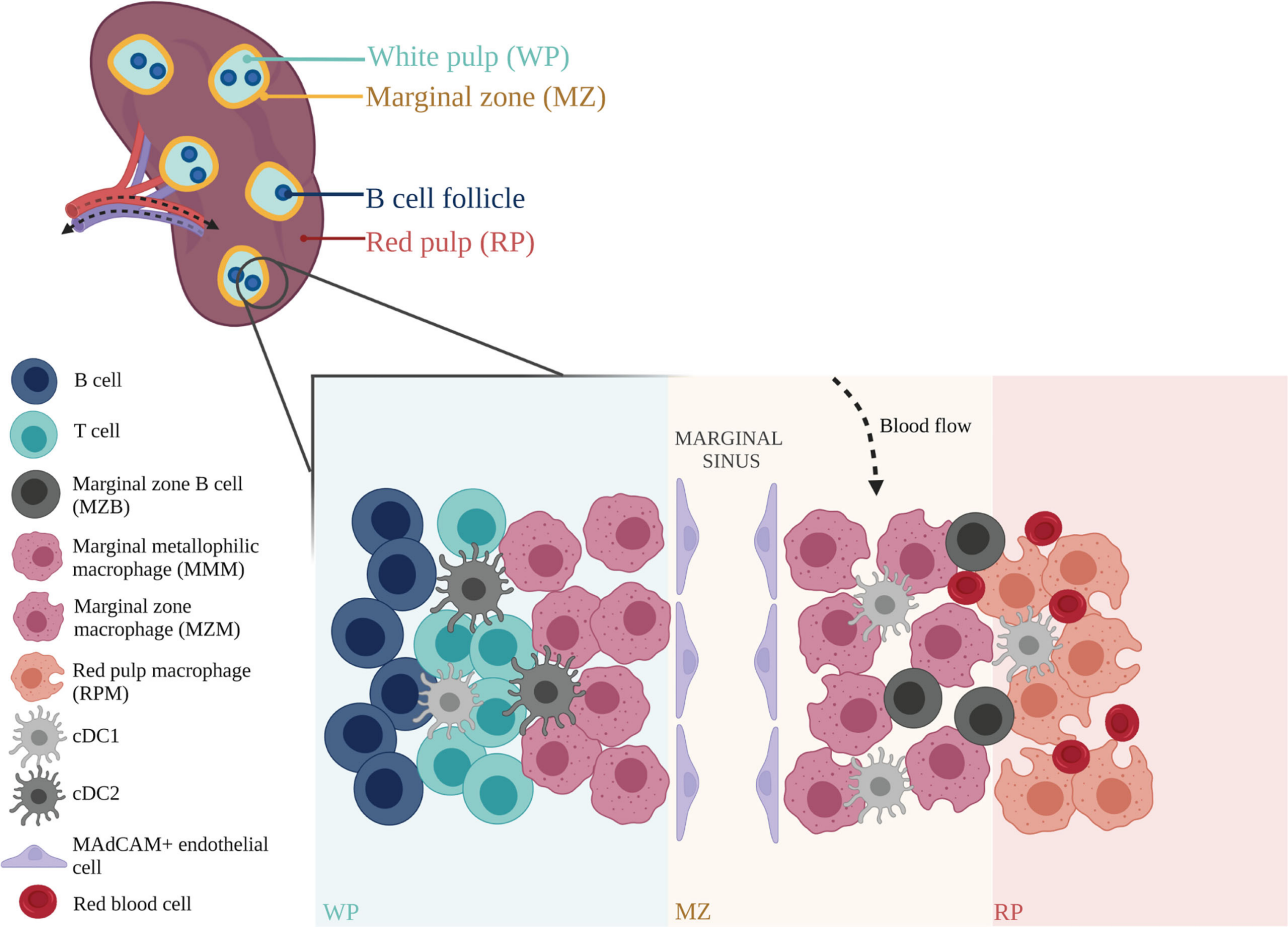
Anatomical structure and organization of mouse spleen. The splenic arterial network functions to filter blood and maintain erythrocyte homeostasis. Arterial blood, arriving at the marginal zone (MZ), passes through the red pulp (RP) cords, and is monitored by red pulp macrophages (RPMs) that survey for blood-borne antigens (78). Blood is recollected in sinuses before exiting the spleen through the efferent vein for return to the systemic circulation (79, 80). Within the spleen, adaptive immune responses to incoming systemic antigens are initiated in the white pulp (WP) which are largely driven by T and B cells (81, 82). Circulating lymphocytes arriving at the spleen may exit the bloodstream and enter the WP *via* the MZ (77). Marginal zone macrophages (MZMs) and marginal metallophilic macrophages (MMMs) are involved in the clearance of apoptotic cells, and maintenance of immune tolerance (83). Their functions are mediated by dendritic cell subsets, cDC1 and cDC2, which present antigens to T cells, and marginal zone B cells (MZBs) that help synchronize immune responses between the adaptive and innate arms (73, 84). This immune network is closely supported by stromal cells such as MAdCAM^+^ endothelial cells that line the marginal sinus, and help mediate tissue homeostasis (77). Figure was created with BioRender.com.

The spleen is composed of the white pulp (WP) and red pulp (RP), with the marginal zone (MZ) situated in between (76, 77), (**Figure 2**). Arterial blood arrives at the MZ and runs through the cords in the RP, where the F4/80^+^ RP macrophages (RPMs) monitor and phagocytose incoming aged erythrocytes (85, 86). In addition, RPMs also extract any dead or opsonized cells from the circulation, while simultaneously surveying for pathogens and tissue damage (78). Other leukocyte populations located in the RP, including neutrophils, monocytes and γδ T cells, exert immune effector functions upon encountering an inflammatory insult (72, 76, 77). Blood is recollected in sinuses to form the venous sinusoidal system and ultimately enters the efferent vein for return to the systemic circulation (77, 79, 80).

The white pulp is the site of lymphocyte differentiation and initiation of immune responses to blood borne antigens. Correct organization and maintenance of the WP is regulated by specific chemokines that attract T cells and B cells and establish their respective zones within the WP (81, 82). The continuous traffic of haematopoietic cells in and out of the spleen is an efficient way for these cells to survey the blood for pathogens and antigens.

The MZ is an important transit area for cells that are leaving the bloodstream and entering the WP. It contains a number of resident cells that have unique properties, including a subset of DCs and innate-like B cells called MZB cells (73, 84). Two specific subsets of macrophages are also found here: MZ macrophages (MZMs) and marginal metallophilic macrophages (MMMs) (76, 87). The MZMs line the outer ring of the WP and are characterized by expression of C-type lectin SIGNR1 and type I scavenger receptor MARCO (85, 88). The MMMs form the inner ring, located closer to the WP (89, 90). These macrophages are characterized by expression of the adhesion molecule SIGLEC1 (CD169) (82, 91–93).

### Structure and Function of the Lymph Node

While the spleen filters the blood and protects the host against blood-borne pathogens, LNs bring antigens draining from tissues together with lymphocytes circulating in the blood. There are more than 20 identified lymph nodes in mice and over 500 in humans, located at multiple sites throughout the lymphatic circulatory system (94, 95). Current understanding of LN structure and function is mainly gained from animal studies.

**Figure 3:**
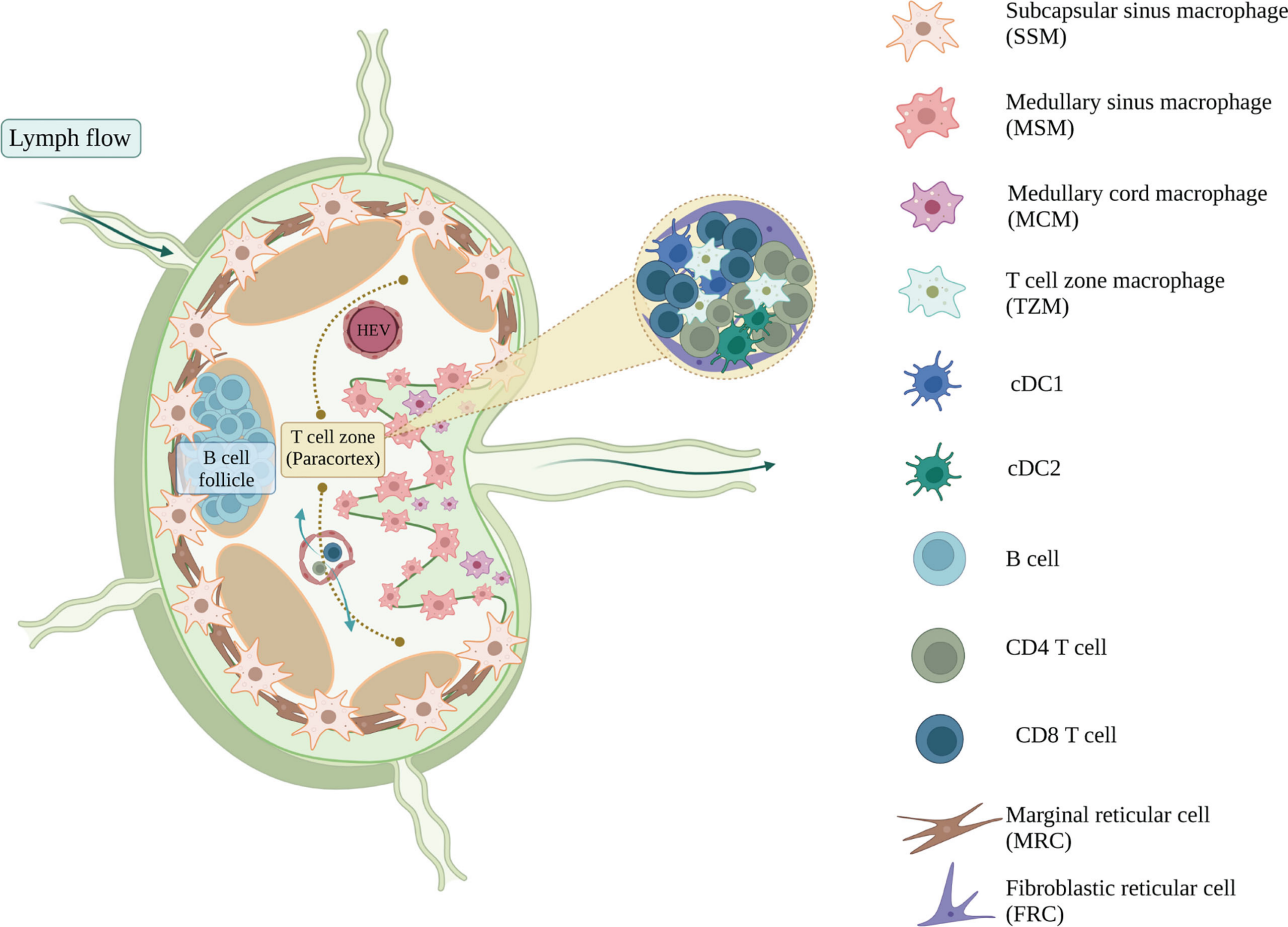
The immune and stromal cell composition of mouse lymph node. Lymph nodes filter the lymph and respond to the lymph-borne antigens, from which T and B cells will get activated, proliferate, and provide adaptive immune responses. Myeloid populations of lymph nodes support and regulate this adaptive response and maintain the homeostasis of lymph nodes (97). Subcapsular sinus macrophages (SSM) survey the infiltrating lymph before they transduce the activation signals towards the B cells sitting in the B cell follicles (98, 99), whereas cDC1 and cDC2 present the antigens that activate the CD4^+^ and CD8^+^ T cells inside the T cell zone, respectively (95). Subsets of lymph node macrophages, including medullary cord macrophages (MCM), T cell zone macrophages (TZM) and tingible body macrophages (TBM), are known for their efferocytotic ability (97, 100). They clear the apoptotic cell debris and maintain the homeostasis of lymph nodes. Lymph node stromal cells also help in regulating tissue homeostasis. Marginal reticular cells (MRC) organize and regulate the B cell follicles, whereas fibroblastic reticular cells (FRC) maintain the T cell homeostasis within the paracortex (101). Figure was created with BioRender.com.

The parenchyma of the LN is compartmentalized by stromal cells and organized into the cortex at the outermost region containing B cell follicles, the paracortex in the inner region containing the T cell zone, and the medulla proximal to the efferent lymphatic vessels containing the medullary sinuses (95, 96), (**Figure 3**). Lymphocytes in the blood enter the LNs *via* high endothelial venules (HEVs) directly into the paracortex area. Lymph, containing soluble and cell-associated antigens, enters the LNs *via* afferent lymphatic vessels and is filtered through the lymphatic sinus (71, 95). Subcapsular sinus macrophages (SSMs) survey and capture lymph-borne antigens before they enter the cortex and paracortex, acting as gatekeepers that protect the host from aberrant immune responses and preventing the systemic spread of antigens (97–99, 102, 103). SSMs initiate innate immune responses by producing pro-inflammatory cytokines to recruit innate immune cells, and can also activate adaptive immunity by extending across the subcapsular sinus floor to present antigen to B cells in the follicles (99, 104).

Lymph passing by the subcapsular sinus will encounter another type of sinus macrophage, known as medullary sinus macrophages (MSMs). MSMs are located along the sinus of the medulla region, interacting with the lymph and also lymphocytes that are egressing the LN (97). Functionally, MSMs can actively phagocytose antigens, as well as apoptotic immune cells in the lymph (105–107). As the lymph passes through the medullary sinus, it then exits the LNs *via* the efferent lymphatic vessel and eventually return to the blood circulation.

The LN contains other macrophages that are also important in maintaining tissue homeostasis and clearing apoptotic debris. Medullary cord macrophages (MCMs) support plasma cell survival and efferocytose apoptotic debris, while tingible body macrophages (TBMs) are involved in the clearance of apoptotic B cells in the germinal centre (97). A recent study has identified a resident macrophage population in the T cell zone, known as T cell zone macrophages (TZMs), that efferocytose apoptotic DCs draining from the periphery (100). As efferocytosis induces an immunomodulatory phenotype in phagocytes, these LN resident macrophages play an important role in immune regulation.

## Apoptotic Cell Clearance and Tolerance in SLOs

Essentially all tissues undergo programmed cell death, known as apoptosis, evident through the constant turnover of cells. Under homeostatic conditions, apoptotic cells rarely accumulate in tissues due to the efficient efferocytosis by tissue phagocytes. The clearance of apoptotic cells is linked to an anti-inflammatory response that also induces immunological tolerance (108, 109). The precise apoptotic cell machinery and phagocytic components involved in apoptotic cell-induced immunosuppression are reviewed extensively elsewhere (109–112). However, it is clear that the efficient clearance of apoptotic cells can be attributed to the redundancy in the mechanisms of apoptotic cell recognition.

Splenic and LN macrophages are known to participate in the efferocytosis of apoptotic cells and maintenance of immune tolerance. TZMs and TBMs in the LN are known to engulf apoptotic debris silently without stimulating T cells, in order to maintain local immune homeostasis (100). The splenic MZ is also involved in the clearance of apoptotic cells from the circulation. IV-injected apoptotic cells drain to the splenic MZ, where they are rapidly engulfed by macrophages in the marginal zone (83). MZMs are known to be critical for particulate trapping in the spleen (72). Depletion studies (in which both MZMs and MMMs are depleted) have also indicated a crucial role for MZ macrophages in apoptotic cell-driven immunomodulation. The initial engulfment of apoptotic cells by macrophages in the MZ is vital in the generation of tolerance to self, whereby delayed clearance of apoptotic cells results in reduced immune tolerance to apoptotic cell-associated antigens (113). For example, depletion of MZ macrophages led to development of systemic tolerance breakdown in mouse models of systemic lupus erythematosus (SLE) and induced inflammatory responses towards apoptotic cell antigens (113).

The infusion of apoptotic cells has been reported to induce immunosuppression in experimental inflammatory diseases, autoimmunity and transplantation. In animal models of autoimmune arthritis, when apoptotic cells were infused not at the joint, but *via* the IV (114–116) or IP route (114, 117), the resolution of arthritic inflammation was conserved at the joint. In mouse models of transplantation, IV infusion of donor apoptotic splenocytes was shown to promote donor-specific immunosuppression, prolonging the survival of heart allografts (118, 119). Clinical studies have also shown that infusion of leukocytes rendered non-viable, either *via* a chemical cross-linker, 1-ethyl-3-(3-dimethylaminopropyl)-carbodiimide (ECDI) or extracorporeal photochemotherapy, is safe and potentially beneficial in multiple sclerosis (120) and cutaneous T cell lymphoma (121). In apoptotic cell-based therapies, the spleen plays a pivotal role as splenic macrophages and DCs are involved in the phagocytosis of apoptotic leukocytes administrated *via* the IV route (122, 123). Together, these findings point to a critical role for the SLOs in the clearance of apoptotic cells and establishment of a tolerogenic state.

## SLOs in Disease

SLOs have a crucial role in host immune defense. In SLOs, antigen priming and immune cell activation occurs, followed by clonal expansion of antigen-specific effector lymphocytes, and the formation of immunological memory and tolerance. The importance of the spleen in host immunity has been demonstrated in various disease settings. In a long-term follow-up study, patients whose spleen had been surgically removed had an increased risk of developing bacterial infections (124). In malaria, the spleen is important in controlling the blood stage infection, clearance of parasitized red blood cells, induction of memory lymphocytes and replenishment of healthy red blood cells (125, 126). Splenectomized patients infected with malaria experienced enhanced parasitic burden, severe disease symptoms, and higher mortality rate (126).

Although SLOs are protective during infection, they may also promote the establishment and progression of some inflammatory diseases. LNs are the key sites for the initiation of GvHD and acute colitis (127, 128). In GvHD, donor cells migrate to the recipient LNs *via* their expression of lymphoid homing molecules, CD62L (L-selectin) and CCR7 (C-C chemokine receptor 7) (129). Upon recognition of alloantigen on the donor cells, T cells in the recipient LNs are activated and migrate to tissues where they cause damage (commonly in skin, gut and liver) (127). In acute colitis, intestinal migratory DCs drain to the mesenteric LNs where they present antigen and induce Th1 or Th17 responses (128, 130, 131). Pathogenic Th17 cells can also migrate from the mesenteric LNs to the gut and cause inflammatory bowel disease (131). Some studies have shown that lymphadenectomy (surgical removal of a group of lymph nodes and surrounding lymphatic tissues) protected rats from GvHD (132, 133). Lymphadenectomy is rarely investigated in clinical studies for the purpose of reducing inflammation, although it is performed on cancer patients to stop the spread of tumor metastases *via* the lymphatic system, or to remove the tumor cells in the lymphatic tissues (134, 135).

The spleen can also promote inflammatory responses in some disease settings. For example, splenectomy showed protective effects (e.g. reduced infarct volume) in rat models of brain, liver, kidney and intestine ischemic injury (136–140). In animal models of ischemic brain injury and also in patients with stroke, spleen atrophy (reduction in splenic weight and size) is commonly observed and thought to be a result of splenic leukocytes egressing *via* the blood circulation and into the injured brain (141). These circulating leukocytes are composed of monocytes, macrophages, neutrophils and lymphocytes, and their migration to the brain exacerbates inflammation and neurodegeneration (142, 143). Splenectomy performed on a rat stroke model (middle cerebral artery occlusion, MCAO), prior to injury, showed neuroprotective effects, with a reduction in brain infarct volume, peripheral immune cells and activated microglia in the brain infarct tissue (140, 142). Apart from the infiltration of splenic immune cells into the brain injury sites, the spleen also contributes to brain inflammation by producing pro-inflammatory signals, such as IFN-γ (140).

The involvement of SLOs and peripheral immune cells in the progression of central nervous system (CNS) inflammation has also been highlighted in other disease models, including spinal cord injury and experimental autoimmune encephalomyelitis (EAE, mouse model of multiple sclerosis) (144–146). In these disease settings, peripheral T cells and myeloid cells are recruited to the CNS and promote disease symptoms by enhancing inflammation. The crucial contribution of splenic T cells in promoting neuroinflammation is further demonstrated in neuropathic pain studies, in which the adoptive transfer of splenic T cells elevated neuropathic pain symptoms in the recipient (144).

Understanding how SLOs regulate the inflammatory response in such disease settings is important in defining the role of these SLOs in mediating the immunomodulatory effects of cell therapy.

## SLOs in Cell Therapy

Given the importance of SLOs in disease progression and tolerance induction, there is emerging evidence for their involvement in cell therapy in experimental disease models. A notable example is stroke, where IV administration of different therapeutic cell types, such as umbilical cord blood cells, haematopoietic stem cells, amnion epithelial cells, bone marrow stromal cells and multipotent adult progenitor cells, were shown to induce neuroprotective effects (147–150). IV-injected therapeutic cells were detected in the spleen and induced an anti-inflammatory environment that promoted repair in the CNS (e.g. infarct size, peripheral immune cell infiltration) (149–151). Of note, a study using neural stem cells to treat stroke showed that the direct interaction between IV-administered stem cells with splenocytes, particularly CD11b^+^ cells, was necessary for the treatment to be neuroprotective, with splenectomy abolishing the beneficial effect (152). In line with this, the improvement in functional recovery from stroke in MAPC-treated animals was also not evident in the absence of a spleen, supporting a critical role for the spleen (153).

### MSC Biodistribution in SLOs

**Figure 4:**
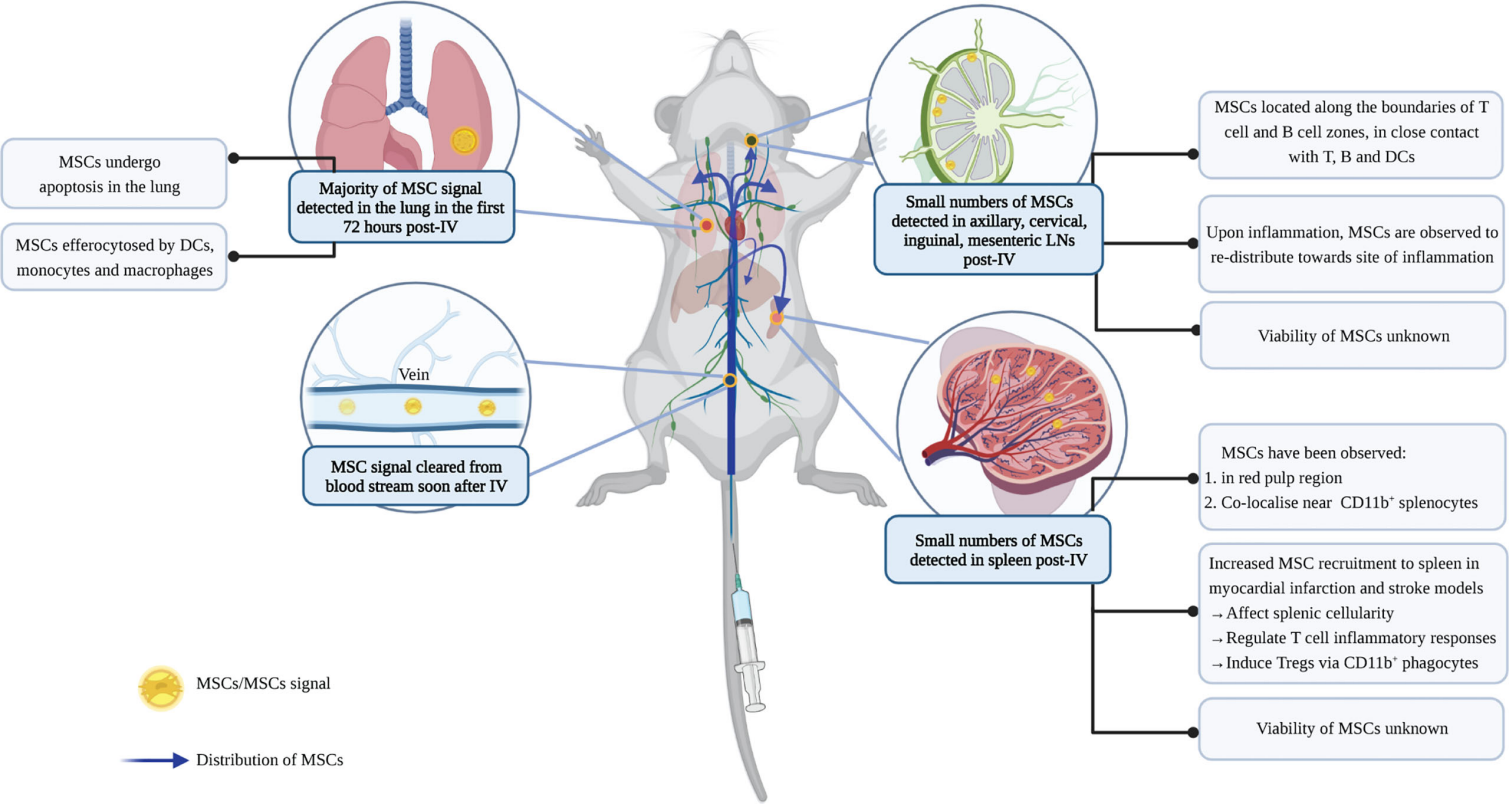
Biodistribution of MSCs post-IV infusion. IV administration is the most commonly used method for MSC delivery. To study the biodistribution of MSCs following IV infusion, studies have used different labelling approaches to track cell signals. Depending on the sensitivity of the techniques, MSCs (or signals of labels) have been found in lung, liver, and SLOs following IV infusion (37, 154). MSCs are cleared rapidly from the blood, and the majority of them are then detected in the lung where they undergo cell death (35). Some signals can also be found in the liver, although these are mostly dead MSCs (37, 154). Dead MSCs in the lung are cleared by lung phagocytes to avoid inflammation (37). Recent studies identified small numbers of MSCs in the SLOs, however, it is unclear whether they remain viable inside the SLOs (154–156). In LNs, MSCs are observed to localize along the boundaries of the germinal center and paracortical area; while in the spleen, MSC signals are detected in the red pulp region, co-localizing with CD11b^+^cells (155, 156). MSCs in the SLOs can regulate T cell response and induce Tregs, and their recruitment is influenced by inflammation. However, the extent by which these immunomodulatory effects in the SLOs are induced by efferocytosis of dying MSCs, or direct contact with viable MSCs or their secretome, remains unknown. Figure was created with BioRender.com.

Despite the central role of the spleen and lymph nodes in the blood and lymph network respectively, MSCs are rarely detected in the SLOs. When reported, the presence of MSCs in the SLOs is usually minimal and requires sensitive detection techniques (**Figure 4**). Small numbers of MSCs were detected in spleen and lymph nodes 24 hours post-IV infusion in naïve mice (154), and for up to 5 weeks in a mouse model of sclerodermatous GvHD (155). Similarly, localization within the spleen and LNs may also occur in the days after IP, but not SC or IM, infusion (44), although MSC biodistribution was variable following this delivery route and attachment of MSC aggregates to the peritoneal walls has been reported (157).

It is thought that the presence of MSCs in SLOs is a result of their active migration, since they express higher levels of homing molecules, including CCR7, CD62L and intercellular adhesion molecule-1 (ICAM-1), compared to other fibroblastic cells (154, 158, 159). MSCs engineered to overexpress CCR7 or ICAM-1 showed improved MSC migration to the spleen and LNs post-IV injection in a GvHD model (158, 159). The increased distribution of MSCs within SLOs correlated with an improvement in their immunomodulatory effects and clinical outcomes in these disease models (158, 159).

The presence of inflammation can influence the biodistribution of MSCs in SLOs. In a myocardial infarction (MI) model, the presence of inflammation increased the recruitment of IV-infused MSCs to the spleen compared to controls without MI (160). In contrast, inflammation can reduce the presence of MSCs in the LNs. In experimental models of colitis and delayed-type hypersensitivity (DTH) (154, 161), accumulation of MSCs was reduced in the LNs and redistributed towards the inflamed tissues.

However, it is important to note the caveats of the labelling approaches used in such studies. For example, membrane dyes are retained when cells lose membrane integrity upon cell death, making it hard to discriminate signals from viable cells, cellular debris, or redistribution by phagocytes that had engulfed apoptotic cells (162). Moreover, studies have repeatedly shown that the small numbers of detectable MSCs eventually get cleared (35, 39, 163), yet their interaction with the host immune system within minutes to hours post-IV infusion is likely critical to therapeutic outcomes. For instance, complement activation by MSCs can adversely trigger IBMIR, but has also been found to upregulate the expression of CD11b on blood myeloid cells, which mediate the immunosuppressive effects of MSCs (164). Clarification of whether the detection of MSC signals in SLOs indicates the presence of viable MSCs or apoptotic cell clearance therefore has important implications for our understanding of the mechanisms of MSC therapy.

### MSCs Regulate the T Cell Profile in SLOs

In several disease settings, the cellular composition of SLOs and function of their immune cells are changed upon cell therapy. The presence of MSCs is also found to associate with a change in the total cellularity of the SLO (154, 158, 160, 165), (**Figure 4**). In a model of myocardial infarction, MSC recruitment to the spleen was shown to decrease splenic natural killer cells and neutrophils (160). In a stroke model, the infusion of human umbilical cord blood cells altered the T cell and monocyte/macrophage composition in the spleen (165). In GvHD and DTH models, MSC recruitment to the LNs was also observed to regulate the survival and activation of lymphocytes, in particular, T cells (154, 158).

The splenic T cell profile is further modulated following cell therapy. Splenic T cells from animals that had received cell therapy were composed of a less pro-inflammatory population (with reduced IFN-γ^+^ and IL-17^+^ CD4^+^ T cells), which exhibited a reduced pro-inflammatory response when restimulated *in vitro* (165, 166). In an autoimmune uveitis model (166), T cells from MSC-treated groups showed reduced proliferative response and produced less pro-inflammatory Th1 and Th17 cytokines, but more anti-inflammatory IL-10, upon antigen restimulation.

Apart from modulating the T cell profile and inflammatory responses, MSC treatment can also induce Tregs in SLOs. In studies of autoimmune disease and allograft transplantation, MSCs inhibited inflammation by inducing an increase in FoxP3^+^ Tregs in the draining LNs and the spleen (156, 166–169). Similarly, an increase in splenic Tregs after MSC treatment was observed in ischemic kidney injury models. The importance of Treg induction in this model was highlighted when the therapeutic effects of MSCs were abrogated following the depletion of Tregs or the complete excision of the spleen (170).

### Myeloid Cells in SLOs Are a Critical Mediator of MSC Effects

Studies have established that co-culturing different myeloid cell populations with MSCs induces regulatory phenotypes, which can modulate immune responses. For example, DCs co-cultured with MSCs downregulated their expression of co-stimulatory molecules and were found to be less stimulatory in activating T cell responses *in vitro* (171–173). Monocytes and macrophages exposed to MSCs were less pro-inflammatory (produced less TNF and IL-12, and more IL-10 and IL-6) (174), more phagocytic and had increased bacterial killing capacity (33, 174–176). Macrophages with an immunoregulatory profile could further maintain an anti-inflammatory microenvironment and influence Treg generation (174, 177). Induction of amphiregulin in MSC-primed macrophages was recently identified as one pathway leading to induction of Tregs and decreased Th1 responses (178). The phenotype and morphology of macrophages ‘re-educated’ by MSCs share some similarities with myeloid-derived suppressor cells (MDSCs), which are differentiated from immature myeloid cells *via* PGE_2_ and IL-10-dependent mechanisms, and have immunosuppressive function (177). However, these *in vitro* findings need to be contextualized within the complex structural organization and cellular composition of the SLOs.

Studies examining immune cell changes in SLOs following MSC therapy support a role for myeloid cells in mediating the immunomodulatory effects of MSCs. When infused in a GvHD model, MSCs overexpressing the homing molecule, ICAM-1, were found in greater numbers in the SLOs (compared to control MSCs) and were found to inhibit splenic DC activation and maturation, suppress CD4^+^ T cell differentiation, and increase the splenic Treg/effector T cell ratio (159). Whilst it remains to be established that the change in regulatory/effector T cell balance is a direct consequence of DC function altered by MSCs in the spleen, studies have linked the induction of Tregs to CD11b^+^ phagocytic cells. Co-culture of splenocytes with MSCs induced Tregs, but not in the absence of CD11b^+^ cells (156). In a model of enterocolitis, the increase in Tregs in the SLOs underlies MSC therapeutic efficacy, which is abrogated upon the depletion of CD11b^+^ cells by clodronate-filled liposomes (156). Importantly, adoptive transfer of CD11b^+^ cells that had been co-cultured with MSCs was sufficient to increase Tregs in the SLOs and protect against disease (156). In another study using an osteosarcoma xenograft model in mice lacking T cells, clodronate depletion of CD11b^+^ splenic macrophages increased the amount of bioluminescence signal of luciferase-expressing MSCs found in the spleen after IV injection, and subsequently facilitated MSC delivery to the tumor (179). The data suggest that MSCs are phagocytosed by macrophages in the spleen, and overcoming this barrier promotes tissue-targeted delivery of MSCs. Thus, while the tolerogenic outcome of efferocytosis can contribute to the anti-inflammatory effects of MSC therapy, settings that require efficient delivery of MSCs to tissues may need to employ strategies that avoid splenic macrophage clearance.

## Conclusion and Future Directions

MSCs exhibit broad spectrum immunomodulatory effects in various inflammatory diseases, but do not persist for significant periods in any part of the body following IV infusion. Instead, their lung entrapment and rapid clearance has shifted the focus to lung phagocytic cells as mediators of their therapeutic effects. Although traces of MSCs have been found in SLOs, along with changes in immune cell composition and function, limitations in labelling and imaging techniques make it difficult to establish with certainty that the small numbers detected are viable MSCs, cell debris or label redistribution following phagocytic uptake. There are several cell types with phagocytic capacity in the SLOs, including migratory cells from the circulation. Cells found in different compartments of the spleen and LNs perform different function, which depends crucially upon their spatial interactions with other cells and chemical cues. Furthermore, SLOs comprise not just immune cells, but also a variety of stromal populations that have functional roles in health and disease (180, 181), including the capacity to efferocytose apoptotic cells (182). Whether these stromal populations have a role in MSC therapy is an open area to explore.

## Author Contributions

DZ, TB, NP, and TH contributed conception and design of the manuscript. DZ wrote the first draft of the manuscript and made the figures. TB, NP, and TH wrote sections of the manuscript. All authors contributed to manuscript revision, read and approved the submitted version.

## Funding

DZ and TB are recipients of the Australian Government Research Training Program (RTP) Scholarship. TH is supported by funding from the National Health and Medical Research Council of Australia (GNT1107188, GNT1162499, GNT2012290) and the Australian Research Council (IC190100026). The Australian Regenerative Medicine Institute is supported by grants from the State Government of Victoria and the Australian Government.

## Conflict of Interest

TSPH received funding from Regeneus Ltd outside of this work.

The remaining authors declare that the research was conducted in the absence of any commercial or financial relationships that could be construed as a potential conflict of interest.

## Publisher’s Note

All claims expressed in this article are solely those of the authors and do not necessarily represent those of their affiliated organizations, or those of the publisher, the editors and the reviewers. Any product that may be evaluated in this article, or claim that may be made by its manufacturer, is not guaranteed or endorsed by the publisher.
